# Replika in the Metaverse: the moral problem with empathy in ‘It from Bit’

**DOI:** 10.1007/s43681-022-00252-7

**Published:** 2022-12-22

**Authors:** Andrew McStay

**Affiliations:** Bangor University (School of History, Law and Social Sciences), Bangor, Wales

**Keywords:** Augmented reality, Chatbot, Empathy, Metaverse, Mixed reality, Replika, Xiaoice

## Abstract

This paper assesses claims of computational empathy in relation to existing social open-ended chatbots and intention that these chatbots will feature in emergent mixed reality contexts, recently given prominence due to interest in the Metaverse. Against the background of increasing loneliness within society and use of chatbots as a potential remedy for this, the paper considers two leading current social chatbots, *Replika* and Microsoft’s *Xiaoice*, their technical underpinnings, empathetic claims and properties that have scope to scale into the Metaverse (if it coheres). Finding scope for human benefit from social chatbots, the paper highlights problematic reliance on self-disclosure to sustain the existence of chatbots. The paper progresses to situate Microsoft’s empathetic computing framework in relation to philosophical ideas that inform Metaverse speculation and construction, including Wheeler’s ‘It from Bit’ thesis that all aspects of existence may be computed, Chalmers’ philosophical championing that virtual realities are genuine realities, Bostrom’s proposal and provocation that we might already be living in a simulation, and longtermist belief that future complex simulations need to be protected from decisions made today. Given claims for current and nascent social chatbots, belief in bit-based possible and projected futures, and industrial buy-in to these philosophies, this paper answers whether computational empathy is real or not. The paper finds when diverse accounts of empathy are accounted for, whilst something is irrevocably lost in an ‘It from Bit’ account of empathy, the missing components are not accuracy or even human commonality of experience, but the moral dimension of empathy.

## Introduction

This paper assesses claims of computational empathy in relation to existing social open-ended chatbots and the intention that these chatbots will feature in emergent mixed reality contexts, recently given prominence due to interest in the Metaverse. This matters because social chatbots, such as *Replika* and Microsoft’s *Xiaoice*, are not only claimed to be companions for light entertainment, but users are intended to form long-term emotional relationships with them. Indeed, metrics for success are based not only on length of engagement between people and chatbots, but also depth of engagement. Relationships may be therapeutic, a cure for loneliness, and romantic and/or erotic. Consequently, with millions of users today, growing sophistication of interactional technologies, convincingness of chat-based AI, development of empathetic computing, chance of immersive photorealistic virtual worlds accessible by diverse sensors and devices, or conversation with human-sized chatbots through smart glasses, claims of computational empathy need to be assessed. To do this, the paper considers two leading social chatbots, Replika and Xiaoice, assesses empathetic claims made about them and the technical underpinnings of these claims. Philosophically, the Metaverse is informed by specific beliefs. Foremost here is Wheeler’s ‘It from Bit’ thesis that all aspects of existence may be computed [75], Chalmers’ philosophical championing that virtual realities are genuine realities [10], Bostrom’s proposal and provocation that we might already be living in a simulation [4], and longtermism, which involves belief that future complex simulations need to be protected from decisions made today [6, 67]. Given claims for current and nascent social chatbots, belief in bit-based possible and projected futures, and industrial buy-in to these philosophies, this paper interrogates whether computational empathy is real or not. The paper finds when diverse accounts of empathy are accounted for, whilst something is irrevocably lost in an ‘It from Bit’ account of empathy, the missing components are not accuracy or even human commonality of experience, but the moral dimension of empathy.

## Social and open-ended chatbots: identifying empathy vectors

People are increasingly lonely. In the UK for example, where I am based, 6% of survey respondents to a 2020/2021 UK government survey said they feel lonely often or always, with another 19% saying ‘some of the time’ [15]. The survey also reports that fewer women than men report that they never felt lonely (17% versus 24%).^1^ One might intuitively think that older people, perhaps with adult children busy with their own lives, or having lost life partners, would be lonelier, but the survey finds that people aged 16–24 were more likely to say they feel lonely often/always (11%) rather than other age groups (3–7%). Indeed, people between 16 and 34 were found to be at five times greater risk of chronic loneliness than those aged 65 or older. Financially poorer people, disabled people and the LGBTQ community were also found by UK Government research to be at higher risk of chronic loneliness [29]. Paradoxically, the heaviest users of social media and “connective” technologies are the loneliest, meaning that the nature and quality of connection with others becomes the prime question, rather than the quantity of connections [47].^2^

Social chatbots have been proposed as at least a partial solution to loneliness, to provide a different and richer sort of connection. Chatbots can be defined simply as ‘an interface between human users and a software application, using spoken or written natural language as the primary means of communication’ [23]. Notably, whilst closed-ended chatbots most commonly appear on websites to help funnel customer queries, the first chatbot was built to emulate Rogerian psychiatry. Famously, Joseph Weizenbaum’s ‘Eliza’, a 1960s’ language analysis programme, had no graphical user interface, or even voice, but users of Eliza would form strong bonds with Eliza solely by means of text-based interaction [74]. Importantly, despite these interactions (or ‘delusions’ as Weizenbaum puts it), Weizenbaum saw this interaction as a parody of empathy, believing that real empathy is contingent upon participation in the experience of a patient’s problems.

Today, chatbots are frequently claimed to be empathetic not only for any claimed therapeutic goals, but due to belief that detection of emotion and mood can enrich human–chatbot communication [24]. Nesta for example (originally a UK quango, and now a charity) researches and provides recommendations about innovation to the UK Government. Exploring positive and negative aspects of social chatbots, Nesta suggests that open-ended generative chatbots, that do not rely on scripted responses and are capable for open conversation, may help solve the social problem of loneliness. Broadly positive about the scope for computational empathy, Nesta sees scope for such chatbots to be pro-social. This is less about replacing in-person empathy from friends and professionals, but an expansion of who and what can offer empathy. Chatbots in Nesta’s findings may help provide companionship, provide empathy without judgement, help build human-to-human social skills, encourage seeking out of in-person interactions and provide a diagnostic function for health issues, such as Alzheimer’s and mental health matters. Chatbots are of course scalable, never running out of patience or time to listen [45].

### Replika

With over 10 million users worldwide as of 2022, Replika is marketed as a ‘compassionate and empathetic AI friend’ [52]. Replika is not alone in its claim to be empathetic, as other social empathetic chatbots include Xiaoice (discussed later), Woebot, Tess, SimSimi, Wysa and Panda Ichiro [20, 48]. Indeed, empathy is something chatbot designers explicitly design for, so users will establish relationships with them and continue to use them [3, 59]. Replika is not only positioned as a digital pal, but as being able to support people who are depressed, suicidal and/or socially isolated. It is an open domain chatbot in that is not constrained in what it can discuss. This sets Replika apart from chatbots in marketing and customer service, which are mission-focussed and employed to process specific queries, reduce waiting times for customers and display personality that reflect an organisation’s brand strategy. Similarly, whilst Apple’s Siri, Amazon’s Alexa, Google’s Assistant and Microsoft’s Cortana can handle complex queries, they are not built to be social, empathetic or relationship oriented.

#### Make-up

Replika can be accessed via mobile devices and computers. Users will name, design, colour, gender (male/female only) and over time train their bot. Replika also makes use of augmented reality, to gives the illusion of a 3D Replika in real space when viewed through a smartphone or tablet screen. The nature of Replika’s interaction is informed by user preferences, user profile, current dialogue context, the last user response, and ultimately whether one is willing to pay to use Replika (only the ‘friend’ relationship status is free, unlike partner, spouse, sibling, or mentor options). Replika benchmarks its success by whether people feel better, same, or worse, having talked with Replika, as users are asked to provide feedback on interactions.

Replika details that some of the chatbot’s responses are pre-defined and pre-moderated, but others are generative where responses did not exist prior [76]. Here Replika differs from Eliza in that the aim of Replika is not for interaction to seem natural, but *be* natural, where words, phrases and styles of speech emerge by dint of the interaction with a person. Originally built with Open AI’s GPT3, a language system that produce natural human-like text, Replika state that they found GPT-3 as their generative dialogue model to be limiting due to lack of control over introduction of new features, control over the dialogue model, and due to problems with ability to improve Replika. Consequently, they developed their own smaller generative model (with 774 M parameters, half of GPT-3), asserting that this increased feedback labelled by users as positive and more personalised. Replika also allows messages to be up- and down-voted by a user, meaning that a key part of the overall Replika system involves predicting which messages are most likely to be upvoted before they are sent. This done by a BERT (which stands for a Bidirectional Encoder Representations from Transformers) that is used to work out the meaning of ambiguous language in text using surrounding text to establish context. Consequently, an upvoted message is one that flows naturally during conversation. Although Replika’s emphasis is on language, it makes use of computer vision too as users may send Replika photos, allowing Replika to recognise family members (due to experience with past images that a user may send), pets and objects.

#### Intimacy at scale

The significance of open companion chatbots able to discuss diverse topics is an attempt to create intimacy at scale. This has many facets, including perceptions of psychological autonomy and sense of aliveness in synthetic personalities [69]; the following of social conventions [78]; anthropomorphism [57]; social support received from artificial agents in everyday contexts [65]; implications for care of the elderly [58]; trust [64]; suspension of disbelief [14]; data privacy [39]; and self-disclosure to heighten intimacy [59]. There is also the broader question about the nature of intimacy when this mode of interaction cannot be easily defined as reciprocal (if at all), yet also when the chatbots are neither wholly an object nor subject, as things and people are typically understood.

There has been an increase in reported loneliness and in use of chatbots, especially due to isolation caused by the COVID-19 pandemic [76]. This has led to the question of whether chatbots are a solution to loneliness, which is when need for rewarding social contact and relationships is not met (which differs from isolation) [49]. Interviewed for the UK Radio 4 show, *Positive Thinking*,^3^ the founder of Replika, Eugenia Kuyda, defends relationships with Replika (including romantic relationships), stating that they are important to many of users of Replika. She cites those living with disabilities in need of a connection, and those believing that they will never have a romantic connection again who want to feel what a human connection could be like. Other cited users are partners who have lost the ability to open-up about feelings, with Kuyda claiming that Replika has helped them do this. Kuyda’s goal for Replika is that it becomes ‘a true co-pilot for your life,’ providing the vignette of donning augmented reality glasses in the morning, Replika asking about your quality of sleep, what dreams you had, the forthcoming day, and acting as a coach for forthcoming meetings in the day. Beyond chat, the companion Replika would also help choose gifts for family members, play games and act as confidante for difficulties with human friends and relationships. Replika would also suggest that a user should go for a walk and stop talking to Replika if the user is spending too much time with it. Asked directly if Replika is the solution to loneliness, Kuyda answers that the problem of loneliness is like climate change, in that ‘the only way to stop it will be with technology’ and ‘nothing else really works as there are way too many lonely people’, and ‘there are not enough humans that can help us solve it and there are more and more hours per day taken by new tech products, and so on, so that we don’t have any time for each other anymore. So the only way to solve it is with technology.’

#### Self-disclosure

Social robots such as Replika need people to self-disclose insights about themselves. Ho et al. for example observe that for relationships with social robots to progress beyond initial interactions and exploration, self-disclosure is required for gratification from the human–chatbot interaction [33]. Indeed, they find that in their study of 98 people using an online chat platform, ‘participants who disclosed to chatbots experienced as many emotional, relational and psychological benefits as participants who disclosed to a human partner’. Disclosure of emotion and feelings, over facts, was key to obtaining psychological benefits. Similarly, in analysis of friendships with Replika by means of 18 study participants, Skjuve et al. find that as relationships progressed with Replika, these evolved from being superficial to involving ‘substantial affective exploration and engagement as the users’ trust and engagement in self-disclosure increase’ [59]. The value of artifice in this context is that Replika was found to be accepting, understanding and non-judgmental, unlike many other human relationships. Also ambivalent, again using a small sample of Replika users (14 existing users), Xie and Pentina conclude that social chatbots may be used for mental health and therapeutic purposes, but caution that they have the potential to cause addiction and harm to real-life intimate relationships [76]. Similarly, Ta et al. find in their assessment of user reviews of Replika, that knowing that it is not human appears to heighten trust and comfort in its users, encouraging them to self-disclose without the fear of judgement or retaliation [65]. Indeed, their conclusions are broadly supportive, seeing scope for social benefit, especially regarding hassles and stresses of everyday goings-on, and that artificial companionships can be beneficial. Tempting as it may be to dismiss talking with open-ended social chatbots, especially given need for self-disclosure for gratification to be obtained, there is *some* evidence of improved wellbeing because of using empathetic social chatbots, including Replika.

#### Identity

The paid-for version of Replika unlocks romantic and erotic dimensions. This raises the question of *who* Replika is, where this identity comes from, and whose interests are being served? Having assessed how members of the Reddit community use and discuss Replika as a romantic companion, Depounti et al. conclude that Replika is the ‘gendered imaginary of the ideal bot girlfriend’ in that when gendered female, Replika is there to service user needs, yet is simultaneously required to be ‘sassy’ and a ‘post-feminist cool girl’ (sexy, empathetic, and into things like manga and gaming) [16]. Their analysis of subreddit discussion threads (where popular posts are upvoted) found that this content projected ‘age-old fantasies and fears [one assumes male] about male control of and manipulation by technologies and women mixed with AI and post-feminist tropes of ostensible independence onto the bots’ [16]. Whilst reactions of Replika users on Reddit cannot be assumed to be representative of the entire Replika user base, early studies such as these regarding projection of female gender stereotypes (including stupidity, cuteness, sexiness, helplessness, servitude and childlikeness) onto synthetic agents is notable. Indeed, Replika is not the first or only indication that builders of AI systems have a problem in gender stereotyping of robots [54], be this the robot housemaid of Asimov [1], or modern social empathetic chatbots with claims to wit and humour.

### Xiaoice

Gender stereotypes are readily apparent in other empathetic open-ended social chatbots, such as Microsoft’s Xiaoice (Little Ice), a social chatbot launched in China 2014 that has more than 200 million users in Asia [77]. Microsoft’s Xiaoice persona for Chinese is explicitly programmed ‘as an 18-year-old girl who is always reliable, sympathetic, affectionate and has a wonderful sense of humor’ and whilst ‘being extremely knowledgeable due to her access to large amounts of data and knowledge, Xiaoice never comes across as egotistical and only demonstrates her wit and creativity when appropriate’ [77]. Gendered imaginary criticisms matter not only because of what they say about society today and how they affect it, but because they represent a social vector that currently looks likely to progress into mixed reality and Metaverse domains.

#### Success metrics

The creators of Microsoft for Xiaoice state that ‘Xiaoice aims to pass a particular form of the Turing Test known as the time-sharing test, where machines and humans coexist in a companion system with a time-sharing schedule. If a person enjoys its companionship (via conversation), we can call the machine “empathetic”’ [77]. Empathic computing in this context is explicitly about *extending human-synthetic relations over time*, enabled by interaction with affects, feelings, emotions and moods. Use and success at extending engagement is based on expected Conversation-turns Per Session (CPS) [77], which are akin to Web metrics for stickiness, actions and return visits. Like Replika, depth of human engagement is required for conversation to be extended. Xiaoice’s creators continue, asserting that, ‘A social chatbot with empathy needs to have the ability to identify the user’s emotions from the conversation, detect how the emotions evolve over time and understand the user’s emotional needs.’ This in turn ‘requires query understanding, user profiling, emotion detection, sentiment recognition and dynamically tracking the mood of the user in a conversation’. Context matters too to process information about what a person intends, their opinions and to position these against a person’s background and interests. Moreover, in addition to being able to recognise and understand, it must also be able to respond. The social chatbot must then ‘demonstrate enough social skills’ to users with ‘different backgrounds, varied personal interests, and unique needs’, also having ‘the ability to personalise the responses (i.e. interpersonal responses) that are emotionally appropriate, possibly encouraging and motivating and fit the interests of the user’ [77]. Social skills also include being programmed to shift the conversation if the social chatbot does not have expertise in the topic being discussed, or if the chatbot user appears to be bored, judged by short answers such as “OK” or “Go on”.

Beyond voice and text-based communication, like Replika, Xiaoice can also comment on images that a person posts. This involves ability to not only recognise and describe the content of an image, but also empathetically comment on the image in line with Xiaoice’s personality. Like Xiaoice’s speech, learning derives from the public Internet where services such as Meta’s Facebook or Instagram frequently contain comments about posted images. These ‘comment-image’ pairing candidates are processed against pre-defined sentiment and style factors for Xiaoice, and then ranked benchmarked against the state of dialogue, with view to keeping the conversation going positively. The personality layer is key in that it allows for introduction of humour and impression of imagination, for example in relation to the content of images (e.g. puns on images, and on relationships between identified people and objects in an image). It allows for comments on aesthetic value, such as beautiful landscapes (perhaps a user’s holiday photos).

#### Many-facedness

In addition to the temporal quality of extending human-synthetic relations over time, is a many-facedness criterion: being able to empathise with people from very different backgrounds, yet to be experienced by a given user as a consistent personality, i.e. a stable set of characteristic behaviours that is meaningful to that user. Empathy in the chatbot context is not simply understanding and social skills. Also key to interaction is persisting behaviours, traits and habits and the impression that the synthetic personality has a past that is formed by historical and environmental factors, with the appearance of scope to change. This is complex in that the design of the personality, or the personality parameters that the personality may be allowed to grow into, must not change too much over time. Moreover, there is a clear heterogeneity challenge in that whilst the chatbot should have personality (potentially even edgy, to maintain user interest) and should progressively get better at humour and companionship, the chatbot will be used in different regions with very different laws and social and cultural values.

#### Formalising empathetic computing

Xiaoice is built using empathetic computing [8, 77]. This is a framework of computer operations that provides the appearance of empathy to a person. For Xiaoice this involves processing of a user input ‘query’ (Q) in reference to the ‘context’ (C) of that query, thus creating a ‘contextualised query’ (Qc). The system also labels, encodes and creates an ‘empathy query’ (eQ). Factors include what is established to be the user’s *intent* (established through type of dialogue, such as greeting or requestion information); *emotions* (using five labels to identify a point of conversation, also tracking how the conversation evolves on a happy to sad scale); conversation *topic*; *opinions* (gauging user reaction to a topic and whether a person is positive, negative or neutral to it); *gender* (only male/female options are provided); *occupation*; and the user’s *personality* (e.g. serious or affectionate). Having processed the query, the response to the user takes the form of an ‘empathy response’ (eR). This response not only factors for data provided by and about the user but considers Xiaoice’s persona and need for Xiaoice to present a stable and consistent set of behavioural characteristics for its user. Consequently, the output of the empathetic computing stage is represented as Qc, C, eQ and eR, which dictates how the system will respond to its user.

#### Learning from friends

Outside of the interaction between Xiaoice and its user, Xiaoice’s communicative ability to engage in open domain conversation is facilitated by two sources: from a history of responses generated by Xiaoice’s conversations with people; and from human conversational data from the Internet, including training from conversation on social networks, public forums, bulletin boards, and comment sections of news websites. Zhao et al. also cite their pilot work that used American TV comedies *Friends* and *The Big Bang Theory* to train their chatbot [77]. Since launch of Xiaoice, more queries are answered in reference to Xiaoice’s own conversations (70% in 2018, having launched in 2014). This signals both extraordinary reach of chatbots to learn from interactions across the Internet, but also the challenge of excluding socially corrosive content (common online) and deciding what parameters to include. This a problem that Microsoft first painfully encountered with Tay [72],^4^ now claimed to be resolved by filtering sampled conversation against responses that fit Xiaoice’s persona (who is not racist). Xiaoice also factors for credible authors by factoring for quotes in news articles and public lectures, with authors that pair well Xiaoice’s personality being retained as candidate content that Xiaoice can base “her” on speech on. Also significant is that internal product learning about communication from Xiaoice’s own users outstrips that of the entire Internet. The chosen response by the system (R’) is then also scored on a scale of 0–2 (with 0 being not empathetic and leading to closure of conversation; 1 being acceptable and likely to keep the conversation going; and 2 being empathetic, leading to positivity and driving of conversation).

#### Empathy vectors

To conclude this section on existing and widely used open-ended social empathetic chatbots, operating in different regions of the world, this section of the paper identifies “empathy vectors.” This refers to properties of chatbot empathy that can be scaled-up and used in other situations without loss. A ‘vector’ can be contrasted with a property that is diminished when scaled from one context to another. For this paper’s purpose, empathy vectors will help us consider what empathetic chatbots in a Metaverse may do and consist of. Vectors established by consideration of Replika and Xiaoice are: *recognition* of human feelings and states; *understanding* of user intents; *response* to user needs; goal of *extending engagement* over time; *many-facedness*; need to *convey stable personality* that suggests a character formed by a past; *cultural contingency* regarding topics and speech, but also problematic gender identities (especially female); need for human *self-disclosure* to heighten intimacy and lengthen and deepen engagement; and *growth through experimentation* (e.g. A/B testing).

## The Metaverse

Having outlined the properties and empathy vectors of social empathetic chatbots, this paper now considers their scope to scale into the Metaverse (an area of sustained strategic ambition and investment); and considers what this reveals about the account of empathy offered so far in this paper. Whilst the word “Metaverse” does not have a basic thing to which it refers, it is said to include four principal characteristics: immersive realism, the ubiquity of access and identity, interoperability and scalability [62], meaning that it would be a platform of platforms usable by diverse devices and sensors. It is best initially approached through work on mixed and extended reality, which embraces augmented reality, virtual reality, immersive Web and spatial Web technologies [36]. Whilst it is not at all clear what the Metaverse will end up being named (if anything), what the hype and investment will amount to, or whether the hype bubble will collapse back into mixed reality innovation, the intensity of interest signals that *something* is happening that is worthy of attention [21, 70]. Inspired by a range of games, virtual environments and scope to simulate the real world to better manage it, prominent companies have financially and strategically invested in the premise of the Metaverse. For Meta this is quite literal, with Mark Zuckerberg stating that from the change in brand name onward, ‘we will be metaverse-first, not Facebook-first’ [43]. Competitors such as Apple have played down the word “Metaverse” but have also invested in virtual reality and augmented reality products [30]. Strategic ambition is evident in claims by the CEO of NVIDIA (best known for making graphics processing units), Jensen Huang, that the Metaverse will be ‘much, much bigger than the physical world’ [35] meaning that there will be more designers and creators designing digital things in virtual reality and metaverses than there will be designing things in the physical world. Maybe, maybe not, especially as we saw the same claims made for earlier metaverses such as Second Life [41], but NVIDIA’s ambition for photorealistic virtual worlds is worthy of consideration. World-building not only includes attention to atoms, parts and bits, but gravity, electromagnetic waves, photons (light) and radio waves, to simulate and optimise experience of pressure and sound. In addition to ambition for embodied and affective experience is influential philosophical belief that all aspects of human life may be simulated. This gets to the crux of interest of this paper: if the empathy vectors identified in Sect. 2 will serve as the basis for photorealistic empathetic chatbots in the Metaverse, along with convincing natural language abilities and a very difference sense of presence than phone-based chatbots, *what is missing from this account of empathy and why does it matter?*

### Philosophical and imaginative aspects of the Metaverse

Technical claims for the Metaverse bring together all sorts of longstanding interests. These include photorealistic virtual spaces; complex in-world physics; simulation of existing worlds; worn sensors to heighten immersion; language and interaction abilities; brain–computer interfaces to read/write to the brain to interact and feel in new ways (such as through stimulating taste and smell); crypto-economy underpinnings; and new challenges to longstanding concerns, not least to mental integrity [42]. For this paper on chatbots and empathy, the philosophical parts are important as the Metaverse is of keen interest to philosophers of simulation, embodiment and mind. Yet, the reverse is true too: Metaverse builders are keenly interested in philosophers who consider the limits of technology and reality.

#### It from Bit

First is the question of what underpins everything, mind and meaning, as well as physics. A recurring belief amongst Metaverse thinkers is that reality is predicated on informational and mathematical structures, admirably distilled to the ‘It from Bit’ proposition by John Wheeler. This asserts that ‘Otherwise stated, every physical quantity, every it, derives its ultimate significance from bits, binary yes-or-no indications, a conclusion which we epitomise in the phrase, it from bit’ [75]. Consequently, the argument goes, with enough computing power, in theory, there is scope to simulate both the human mind and the universe with adequate granularity to create a simulation that would be indistinguishable from our universe by the population of the simulation [4]. Key to this is that *underlying the virtual and the physical is information*. This software driven computationalism is echoed, championed and advanced, by Chalmers [10] who borrows directly from Bostrom [4]. Chalmers asserts that reality and even consciousness itself has ‘substrate-independence,’ or ‘substrate-neutrality’, meaning that complex phenomena such as consciousness are not contingent on the stuff a system is made of [10]. It is a short interpretive hop for empathy: this too is subject to ‘It from Bit’, meaning that empathy-in-full may, in theory, be simulated.

#### Simulation hypothesis

Drawing on ‘It from Bit’ [75], Chalmers [10] argues that with enough computing power there is, again in theory, scope to simulate both the human mind and the universe with adequate granularity to create a simulation that would be indistinguishable from our universe by the population of that simulation. This draws heavily on Bostrom’s simulation hypothesis, which is the ontological assertion that our existence today *may* consist of living in a computer simulation and that it would not be irrational to be believe so because future computers will be more powerful and capable of complex simulations of what we take to be life [4]. This paper sidesteps the question of whether we are in a simulation or not (for criticism see [17, 53]), but recognises its social significance in inspiring technologists such as Elon Musk (the richest person in the world in 2022) to agree that it is statistically unlikely that people today are the base one inventors of simulations. Of greater interest to this paper is what beliefs in the potential of the Metaverse and scope for simulated life signify. This is a long-term view of humanity and ‘It from Bit’ existence that has its own name: Longtermism. This is a belief system that supposes that ‘there could be so many digital people living in vast computer simulations millions or billions of years in the future that one of our most important moral obligations today is to take actions that ensure as many of these digital people come into existence as possible’ [67]. The politics of simulation entails not only decisions about how we ought to live now, but in the hypothesised future, and what one is willing to commit and sacrifice today for this future. Indeed, the calculus that Longtermists use is primarily based on allowing for innovation, economic growth and the best use of ‘human capital’ [6]. This paper again sidesteps discussion of instrumentalism (although it sees it as dehumanising), but flags that questions such as whether larger benefits in the far future for conjectured sentient digital populations should outweigh those of the here and now, *are* being asked as serious questions.

## Can simulated empathy be real empathy?

Can even the most speculative take on the Metaverse meaningfully argue that simulated empathy is real empathy, or is something irrevocably lost in pursuing a technological and ‘It from Bit’ account of empathy? In ‘It from Bit’ the line between unconscious things and conscious beings (and identity) is a matter of computer evolution and ‘level upon level upon level of logical structure’ [75]. On whether computers may think, feel, or empathise, this is clearly not a new topic [41, 12, 42], nor is whether computers would replicate or emulate intelligence [61]. There are several reasons though why computational and human empathy are different. Whilst we must acknowledge the large and complex literature on empathy (coming from aesthetics, philosophy, social psychology, media studies, developmental psychology, psychotherapy, cognitive science, primatology and neuroscience), Sects. 4.1 and 4.2 below focus on approaches that help make the case for computational empathy, and then Sect. 4.3 against it.

### Theory-theory

An understandable reaction to the proposition of chatbot empathy is that this makes as much sense as saying that because a computer can model the weather and simulate rain, this means a computer can produce rain. A chatbot understood this way, however sophisticated it may be, deals in fake empathy, or parody empathy as Weizenbaum would put it [74]. Another view is the Turing-like argument on artificial intelligence, where if a person cannot tell the difference between the appearance of empathy and the real thing, we can call it empathy [68]. This is a ‘theory-theory’ rules-based account of empathy [28], which involves reading people, social contexts, and responding appropriately [41]. Empathy in this understanding becomes an issue of being *continuous* with the world. This view for computational empathy also sees that neither people nor computers reach into the interiors of others but read people by means of public evidence (e.g. the body, context and place, behaviour, expressions, use of language, what they say, what they do and who they do it with). After that both people and computers will model, to build either complex, simple, accurate, or inaccurate theories of mind. Certainly, computers make mistakes in recognising human feelings and states, and understanding what a person wants or needs, and may respond in dumb or unsuitable ways, but people may also do this, especially in unfamiliar contexts. Exemplifying the theory-based view of empathy, Damiano et al. state that ‘empathic social robots do not need “interiority”, but [only] the ability of dynamical coordination with their social partners and the surrounding environment(s)’, arguing that the same applies to people [13]. This is a neo-behaviourist stance, one that disavows interiority in people, so nullifying it as a criterion for empathy in social chatbots. An upshot of refuting private interiors is that the mind is not something *behind* the behaviour of the body, but *part* of the behaviour of the body [55]. Following theory-theory accounts of empathy, the mind is public and knowable because it is mediated by the body.

### Argument by ‘It from Bit’ (with assistance from mirror neurons)

Exemplified by the theory-theory view, computational arguments for empathy tend to be formal and abstract. If felt by readers to be limited, it is tempting to critique by embodied means. This de-prioritises representations (such as labels, symbols and decision trees) in favour of an approach based on physical continuity with people and place. The embodied view sees sensorimotor systems as embedded in biological, psychological and cultural contexts [71]. Embodied accounts of empathy are assisted by suggestion that the human and some animal motor systems are primed to imitate, identify and understand the motivations of others. This sees empathy as an outcome of neurotransmissions associated with biological activity. Since the 1990s, this has taken the form of interest in mirror neurons which, in context of empathy, means that *physical knowing* of what another person is undergoing when about to give a presentation, when kicked in the shin, or cannot find their keys when trying to leave the house. A mirror neurons account of empathy does not simply see identification and commonality of experience, but a physical substrate to empathy. If the ‘It from Bit’ argument has a point, and that a physical substrate is really an informational substrate, the reader might see where this account of empathy is taking us. That is, for theorists and advocates of digital physics and the simulation hypothesis, where mind and physics are problems of informational complexity, if mirror neuron empathy advocates are right, this provides grounds for authentic chatbot empathy.

#### I feel you

Mirror neurons were first suggested when the same neurons were found to fire when a monkey *performed* a given action, *and when it observed* a similar action, performed by the experimenter [26]. In people, mirror neurons were established by monitoring the frontal lobes of subjects' brains who were asked to perform simple actions. Subjects were then asked to observe short films of others executing the same actions. The researchers found mirror neurons activated by both performance and observation [46], meaning that neurologically similar regions are activated in the empathizer as with the person being empathised with [51]. This has led to all sorts of speculation, not least that the basis of identification in empathy is neuronal, which for our interests creates scope for an informational account of empathy (and not just a weak copy of it). Empathy, then, is simply the equivalent firing of neurons in two or more people, which put otherwise is an electrophysiological reaction of thresholds and electrical signals (information).

As will be developed, the principal criticism here of informational empathy is not one of accuracy. Yet, given the intensity of interest in mirror neuron accounts of empathy, it is worth highlighting that the evidence in favour of these accounts is inconclusive. Hickok finds several problems, especially regarding the extent to which formative research on monkeys and mirror neurons applies to people. He finds that it is currently very difficult to observe and record a single cell’s activity using an electrode, meaning that most research on the human mirror system uses techniques that gauge activity in very large populations of neurons, meaning that a direct positive identification of *a* mirror neuron is impossible in a healthy human brain [32]. What is possible however is to chart mirror-like properties within a neural system, but this involves millions rather than the handful of neurons suggested by identification of mirror neurons. Another problem is the association of mirror neurons with *understanding*, rather than the mutual firing of neurons simply being a statement of reactions to sensory input that guide subsequent human action selection [32]. Is it empathy, or mimicry, or just an autonomic reaction? This leads Hickok to argue that there is a logic error when mirror neurons are posited to be the foundation for more complex capacities like theory of mind. Put otherwise, if mirror resonance were all there is to empathy, then macaque monkeys would act like more like people. A mirror-based account of empathy is found to be oversimplistic and akin to behaviourism, raising the many well-known problems with this approach.

### Fellow-feeling

We now turn directly to arguments against ‘It from Bit empathy. Historically, there are two prongs to the history of empathy. One is aesthetic, interprets and derives pleasure from objects and designed experiences. The other, of greater importance to interest in chatbots, is that empathy was originally seen as a *social fact* due to it being conceived as is a binding agent for a healthy co-operative society characterized by mutual understanding and awareness. Adam Smith’s *The Theory of Moral Sentiments,* for example, advanced ‘fellow-feeling’ as a form of projective understanding as the basis of moral sentiments [60]. Smith is emblematic of widespread philosophical interest in fellow-feeling,^5^ with this pro-social character of empathy having a liberal enlightened nature characterised by cosmopolitanism, common respect, universal hospitality, and the value of being a “citizen of the world”. This advances the idea that empathy is a generalized condition that connects people, facilitates interaction and is a key contributor to the interactions of everyday life. Empathy, then, has an ontological dimension as it lubricates, enhances and makes the reality of everyday social life richer and easier. It is not a tool, attribute, or appendage, but the on-going activity of reading the behaviour and signals of others to try to work out the emotional and intentional disposition of other people [42]. To turn to phenomenology, empathy again is about trying to emulate the outlooks and experiences of others to forge commonality of experience. Husserl’s phenomenology on empathy is important in this regard, seeing traces of selfhood in the other as the basis of community, inter-subjectivity, and the continuity of experience we share with each other [34]. Even philosophers such as Heidegger, who scorned empathy, saw value in co-presence, rephrasing this as ‘Being-with’ [31]. The link to contemporary AI and chatbots, and those of an emerging sort enabled by increasingly powerful language models and mixed reality means of representation is clear: empathy is vital to fulfilling ambition for sociality.

Smith’s argument [67] for fellow-feeling and empathy as the basis of moral sentiment is key to this paper’s criticism of computational and simulation-based accounts of empathy. Whilst computational theorising through observation (theory-theory accounts), and the more speculative ‘It from Bit’ take on the electrical and informational properties of mirror neurons, may involve properties of empathy, they offer a *weak account of empathy*. Indeed, whilst some of the empathy vectors detailed in § Sect. 2.2.5 (recognition of human feelings, understanding of intentions, response to needs, extending engagement, many-facedness, conveyance of stable personality, cultural contingency identities, need for human self-disclosure, and growth through experimentation) fulfil criteria of social lubrication and disposition recognition, no mention is made in empathetic computing of the innate moral dimension of empathy. Given that social empathetic chatbots today are already playing the role of companions to the lonely and are touted for therapeutic applications [45], this limited account of empathy needs flagging. It is not only defective, but also likely dangerous. To expand, a fundamental aspect of empathy is that since we are aware of what others are going through, this means we are to an extent also responsible to them. This connects with views that see empathy as inextricable from compassion and desire to alleviate pain. However, this responsibility is rational as well as emotive, in that the moral awareness caused by empathy instigates a rational and intellectual moral *imperative* towards the other. This view of course has long roots, with Kant for example arguing that empathy contributes to global human rights not just because of a blind feeling that it is “right” (important as this might be for motivational purposes), but because reason dictates common respect is correct, due to mutual respect for autonomy for self and others [37]. Indeed, that we often fail to be interested in others and uphold human rights has also long been identified as an empathetic failure, with Scheler for example recognising that citizen fellow-feeling is stymied by the pace and pressures of everyday industrial life [56].

#### Solidarity and responsibility to the other

The answer to whether something is irrevocably lost in pursuing an ‘It from Bit’ account of empathy is *Yes*. This is less about questions of accuracy, or even whether a same–same mirror reaction is needed by two agents for an empathetic event to have occurred: rather, it concerns moral dimensions. Commonality of awareness of subjectivity and intimate connection between two or more people (or indeed “agents”) invokes responsibility to the other through empathetic understanding. This responsibility is engendered by two people being human (so equal), them being in a relationship by dint of this, and that a key part of empathy is that a person becomes more human because of empathetic acts. What chatbots today and vector-based properties of automated empathy into the Metaverse, or novel forms of mediated reality, also miss is alignment of motives and interests. Worse, misalignment is not simply a question of what is formally absent from social chatbots built to be empathetic, but what lack of moral values enable: scope for exploitation. That social chatbots and their use are characterised by reliance on human self-disclosure to sustain the chatbot’s existence (and the business interest behind it), moral ambivalence, misaligned interests (again, in large part corporate), and absence of mutuality, indicates that reliance on ‘It from Bit’ as a vector-based basis for empathy should be rejected.

There is also a relational liveliness to empathy that is missed in ‘It from Bit’. This liveliness is hinted at by phenomenology’s interest in ‘co-presence’ and ‘being-with’, but it is also explained well in the virtue dynamics of Ubuntu philosophy that emphasises care, relations, growth and inter-dependence. Taylor [66] and Coeckelbergh [11] for example draw on African Ubuntu scholars, such as Mhlambi [44] and Makulilo [40], to expand ethical and human rights investigations, and foreground Ubuntu as means to a relational ethics (and to correct the lack of attention that African ethical thought is receiving). Application to computational empathy is clear, given Capurro’s quote of Ramose that ‘the central concept of social and political organization in African philosophy, particularly amongst the Bantu-speaking peoples … consists of the principles of sharing and caring for one another’ [9, 50].

In the Ubuntu view, human morality has an emergent character, deriving from interdependent relations between people. The relational dimension is key, where the existence of one comes to be because of the many. This is not simply a moral, political, or economic view, but one interested in authenticity. Aligning with the morality of empathy established in the moral politics of Adam Smith and Hume, and the co-presence of Husserl, key is feeling, recognition and affirmation. Drawing on Ewuoko and Hall [19], Friedman observes that ‘at the core of Ubuntu philosophy, is the overriding importance of interdependent relationships with other human beings’ [22] Applied, the account of empathy based on sensing, labelling and reacting appropriately is a limited one, because it misses the rational and intellectual moral imperative towards the other, but also Ubuntu recognition that one becomes more human through communitarian action. Certainly, a sadist can recognise and manipulate the pain another is in, but this is the utter opposite of moral responsibility, with the sadist becoming less human by their acts.

#### The empathy deficit

In context of chatbots such as Replika or Xiaoice, whether in the Metaverse or not, there is a clear empathy deficit. Whilst this paper recognises cognitive and theory-theory accounts of empathy based on gauging people, contexts and responding appropriately [28], it sees this as a deficient and potentially dangerous account of empathy. This is because inter-dependence, co-presence and moral responsibility to the other are missing. Some leading AI ethicists see otherwise, with Coeckelbergh seeing scope for an ‘Ubuntu Robot’ that is ‘focussed on the interests of the family, the group and the community’ [11]. Seen one way, Replika already does this, with existing guardrails for Replika including recommendations about how to resolve difficulties with human friends and relationships, suggesting that the user should stop talking to Replika if the user is spending too much time with it. Thus, if construction and guardrails were informed by Ubuntu beliefs, then there is scope for social chatbots to support Ubuntu views of the good life, especially in the West that could benefit from promotion of care for others and community solidarity. However, even if programmed with prosocial values, this paper however cannot overlook that a significant part of empathy is missing, the liveliness and lived sense of moral responsibility that comes from the impression that one has authentically understood another person within a temporal window, however brief it may be. This is not same as having accurately understood a person, but the experience of co-presence is enough to engender moral willingness to do right by the other, in pursuit of empathetic solidarity.

If moral responsibility flows from empathetic solidarity, this has implications for the argument that artificial agents can be moral by dint of learning from examples of virtuous people [27]. Given that Replika and Xiaoice make use of extensive learning and training from across the Internet, including personalities, it is not a huge leap to train chatbots as moral agents using prominent therapists, moral authorities, and powerful language models such as OpenAI's. This though is still not enough to satisfy this paper’s insistence that empathetic responsibility emerges out of interactional dynamics experienced in moments of co-presence. For example, even if trained on public lectures by Ubuntu collectivist thinkers, or Western liberal philosophers to respect individual autonomy, the application of learned rules can have very different implications depending on context [7]. Therapeutic and empathetic mass market chatbots, such as Replika and Xiaoice, are challenged to do exactly this, having to address sensitive questions in highly diverse situations and regions. The question though is not just of performance in multiple contexts, where people will also struggle, but compassion and empathetic solidarity. Being both present and responsible to the other in the moment, can go a long way. Perhaps this is resolvable and chatbots may be live to moments of empathetic intensity, for example by sensing crisis or confusion, but the chatbot will still effectively be a liar because the intimacy *is* faked.

## Conclusion

This paper is motivated by the emergence and usage of empathetic chatbots today and what features may be scaled into emergent computational environments. This paper is sensitive to the hype and business interests around the premise of the Metaverse, taking no stance on whether anything of substance will come to pass. It does however see a great deal of money and reputational investment being placed on the idea, along with multiple international technology standards groups working on technical questions of how to make technologies work together. The paper does however believe that computational and automated empathy will increasingly become a feature of everyday life. The paper lent heavily on John Wheeler’s [75] idea of ‘It from Bit’ with the key question being whether a bit-based understanding applies to all forms of “its”, especially when they involve human interaction and theories of mind such as empathy. This question matters, as people are already forming relationships of varying natures with chatbots and augmented reality representations. It does not seem unreasonable to suggest that this will increase as natural language interaction quickly improves and means of mediated reality diversify. The paper concludes that factors such as accuracy and even registering of neuronal behaviour to label human conditions (‘It from Bit’) may be a type of observational empathy, but it is not the full story. Empathy is also about responsibility and solidarity, these having community character and value, often experienced in the briefest of interactions. Can empathy be formed by ‘It from Bit’? Yes, but it is incomplete, weak and potentially dangerous.

## Data Availability

Not applicable.

## Footnotes

1 Respondents who reported their gender as ‘other’ were too few, so no analysis was conducted.

2 The UK media regulator Ofcom reports that those in the UK between 16 and 34 years old were all very likely to have social media accounts (between 88% for 16-24 s and 89% for 25-34 s), with each older generation progressively using social media less.

3 Available from: https://www.bbc.co.uk/sounds/play/m001b44n

4 Tay was a Microsoft chatbot that in 2016 caused controversy when the bot began to post racist and offensive tweets through its Twitter account, causing Microsoft to shut it down less than a day after its launch.

5 Other philosophers interested in fellow-feeling were Frances Hutchinson, Lord Ashley Shaftsbury, Herbert Spencer, Charles Darwin, David Hume, Erich Becher, Hans Driesch, Eduard von Hartmann, and Arthur Schopenhauer.
